# Exterminate the Pests

**Published:** 1916-02

**Authors:** 


### Exterminate the Pests.

It is an old theme and may appear a trifle monotonous to physicians, but upon them largely devolves the responsibility for creating public sentiment that will result in the extermination of the fly and the mosquito. It will be too late next summer to begin an agitation that will prove effective. If large results are to be obtained, work must begin early and kept up until late. The people expect physicians to lead in all campaigns for the improvement of health conditions, and they have a right to expect it. The common fly is the most dangerous of all disease carriers, and its activities are ceaseless.

Without running any risk of being accused of self-exploitation, physicians can use their local papers to direct attention to the dangers from flies, and can urge that steps be taken to exterminate them. The papers will be glad to publish all such articles when they come from reputable local physicians, for there is not a newspaper anywhere that is not anxious to do its part to advance the health of a community. The only reason they do not have more to say on such subjects is either that they do not realize the full importance of it, or else they do not know just how to present it. Most of them do have occasional articles about the fly as a disease carrier, but they are not sufficiently frequent or informational enough to effect results. Besides, the people pay more attention to what a physician says about health measures than to what others may think of them.

Impress upon the community that flies breed only in filth, and that in civic and private cleanliness alone can the health of a community be improved. Then call attention specifically to the kinds of places where flies breed, and show that those places exist in every town and about almost every home. So many people look beyond their own doors to find filth when they unknowingly have it all about them. Show local officials, as well as individuals, what they can do to conduct an effective campaign against the fly and mosquito. In some localities mosquitoes have been entirely eliminated and flies have become scarce indeed, but these results have been obtained only where there has been concerted effort, and in every instance where some public-spirited physician has taken the time to lead.

But it will be too late to begin such a campaign next summer after an epidemic of some kind has done its worst, or after the flies and mosquitoes have multiplied by the thousands. This is one case in which all people can agree that preparedness is the only means of averting danger. The time to prepare is now.

